# Editorial Note: A nonenzymatic dependency on inositol-requiring enzyme 1 controls cancer cell cycle progression and tumor growth

**DOI:** 10.1371/journal.pbio.3003920

**Published:** 2026-07-29

**Authors:** The PLOS Biology Editors

S3 Fig in [1] is a duplicate of S1 Fig. The correct S3 Fig is provided with this notice. The publisher apologizes for the error.

During post-publication discussions, the corresponding author stated that some of the blot panels presented within a figure in [1] were run on different gels and blots. Specifically, the following figures show proteins run on different blots, with the total number of different blots in each figure in parentheses: Fig 3H (2), Fig 3I (2), Fig 6B (6), Fig 6D (4), Fig 7A (2), Fig S1A (2), Fig S1D (2), Fig S1F (2), Fig S1I (2), Fig S1L (2), Fig S2C (3), Fig S3A (2), Fig S3H (2), Fig S3J (4), Fig S3L (2), Fig S3M (3), Fig S5C (2), Fig S5F (2), Fig S5G (2), Fig S6A left (5), Fig S6A right (4), Fig S6B left (4), Fig S6B right (4), Fig S7A (2). The corresponding author stated that within each of these figures, a single master set of samples was used, and they provided a schematic illustration of the blotting procedure (S1 File). These samples were normalized by buffer addition for total protein content as measured by BCA assay, simultaneously boiled in denaturing buffer, and loaded in equal volume onto several gels. Identical electrophoresis and electrotransfer conditions were used for all resulting gels and blots, which were also stained with Ponceau-S to visualize total transferred protein.

In addition, there are errors in the Funding and Competing Interests statements. The correct statements are:

Funding: Genentech, Inc provided support for this study through salaries for IZG, DL, IO, SM, XPJ, CJG, DK, ES, KC, MB, MGB, JR, ZM, MC, WS, MR, DCD, and AA and through funding for laboratory research. The funder had no additional role in study design, data collection and analysis, decision to publish, or preparation of the manuscript.

Competing Interests: The authors have read the journal’s policy and have the following competing interests to declare: IZG, DL, IO, SM, XPJ, CJG, DK, ES, KC, MB, MGB, JR, ZM, MC, WS, MR, DCD, and AA were paid employees of Genentech, Inc at the time the study was conducted. There are no patents, products in development, or marketed products to declare. This does not alter the authors' adherence to PLOS *Biology* policies on sharing data and materials.

The *PLOS Biology* Editors issue this Editorial Note to inform readers of the above information and to provide the correct S3 Fig.

## Supporting information

S3 Fig IRE1 silencing downregulates cell cycle genes and engages TP53 and specific CDK inhibitors. (A) RNA sequencing sample validation. AMO1 shIRE1 cl.1 or cl.3 cells and shXBP1 cl.1 and cl.18 cells were incubated for the indicated time with Dox (0.2 μg/ml) in triplicates and analyzed by IB. **(B)** Cells as in A were analyzed by RT-qPCR. Data points represent one biological replicate. Error bars represent SD. **(C)** Effect of IRE1 or XBP1 knockdown on mRNA expression of select XBP1s- and RIDD-target genes. Cells as in A were analyzed by bulk RNA sequencing (RNAseq) for mRNA expression of the XBP1s targets *Sec61A1* and *SYVN1* or the RIDD targets *DGAT2* and *BCAM*. **(D)** Row-clustered heatmap of the scaled mRNA expression by bulk RNAseq of genes involved in the S phase of the cell cycle from RNA sequencing of cells as in A, used to calculate the S phase score in Fig 3B. **(E)** Row-clustered heatmap of the scaled mRNA expression by bulk RNAseq of genes involved in the G2 and M phases of the cell cycle from RNA sequencing of cells as in A, used to calculate the G2/M phase score in Fig 3C. **(F)** PROGENy pathway analysis of the effect of IRE1 or XBP1 knockdown on mRNA expression. PROGENy scaled pathway score heatmap depicting p53 as one of the most differentially altered pathways upon IRE1 versus XBP1s knockdown for samples depicted in Fig 3A. **(G)** Effect of IRE1 or XBP1 knockdown on mRNA expression of p53 target genes. Row-clustered heatmap depicting scaled mRNA expression by RNA sequencing of the top 100 TP53 pathway response genes for samples shown in Fig 3A, given the PROGENy model. **(H)** Effect of IRE1 or XBP1 knockdown on p53 cleavage. AMO1 shIRE1 cl.1 or shXBP1 cl.1 cells were incubated for the indicated time with Dox (0.2 μg/ml) and QVD (30 μM) and analyzed by IB. Representative blot of 3 independent experiments shown. **(I)** Effect of IRE1 or XBP1 knockdown on CDKN1B/p27 protein levels. Data depicted are from the proteomics analysis described in Fig 3F. Data points are mean ± SE for all biological and technical replicates normalized to *t* = 0 h for each cell line. **(J)** Effect of IRE1 knockdown on p53, p21, and p27 protein levels. AMO1 shIRE1 cl.1 and KMS27 shIRE1 cl.9 cells were incubated for the indicated time with Dox (0.2 μg/ml) and analyzed by IB. Representative blot of three independent experiments shown. **(K)** Effect of IRE1 or XBP1 knockdown on p27 mRNA levels. AMO1 shIRE1 cl.1 or shXBP1 cl.1 cells were incubated for the indicated time with Dox (0.2 μg/ml) and analyzed by RT-qPCR. Data points are one biological replicate normalized to its untreated counterpart. Representative plot of 3 independent experiments shown. **(L)** Effect of IRE1 or XBP1 knockdown on H2AX and phosphorylated (γ) H2AX (gH2AX). AMO1 shIRE1 cl.1 or shXBP1 cl.1 cells were incubated for the indicated time with Dox (0.2 μg/ml) and analyzed by IB. Representative blot of 3 independent experiments shown. **(M)** Effect of IRE1 or XBP1 knockdown on p53, p21, and 53 BP1 proteins. AMO1 shIRE1 cl.1 or shXBP1 cl.1 cells were incubated for the indicated time with Dox (0.2 μg/ml) and analyzed by IB. Representative blot of three independent experiments shown. All raw data can be found in S1 Data.
(TIFF)

S1 File Immunoblotting layout. This diagram was also published in [2].
(PDF)
