# Supplementary figures and images for: Editorial Note: A nonenzymatic dependency on inositol-requiring enzyme 1 controls cancer cell cycle progression and tumor growth
Source: PLoS Biol. 2026 Jul 29;24(7):e3003920. doi: 10.1371/journal.pbio.3003920 (PMC13419169; doi:10.1371/journal.pbio.3003920)

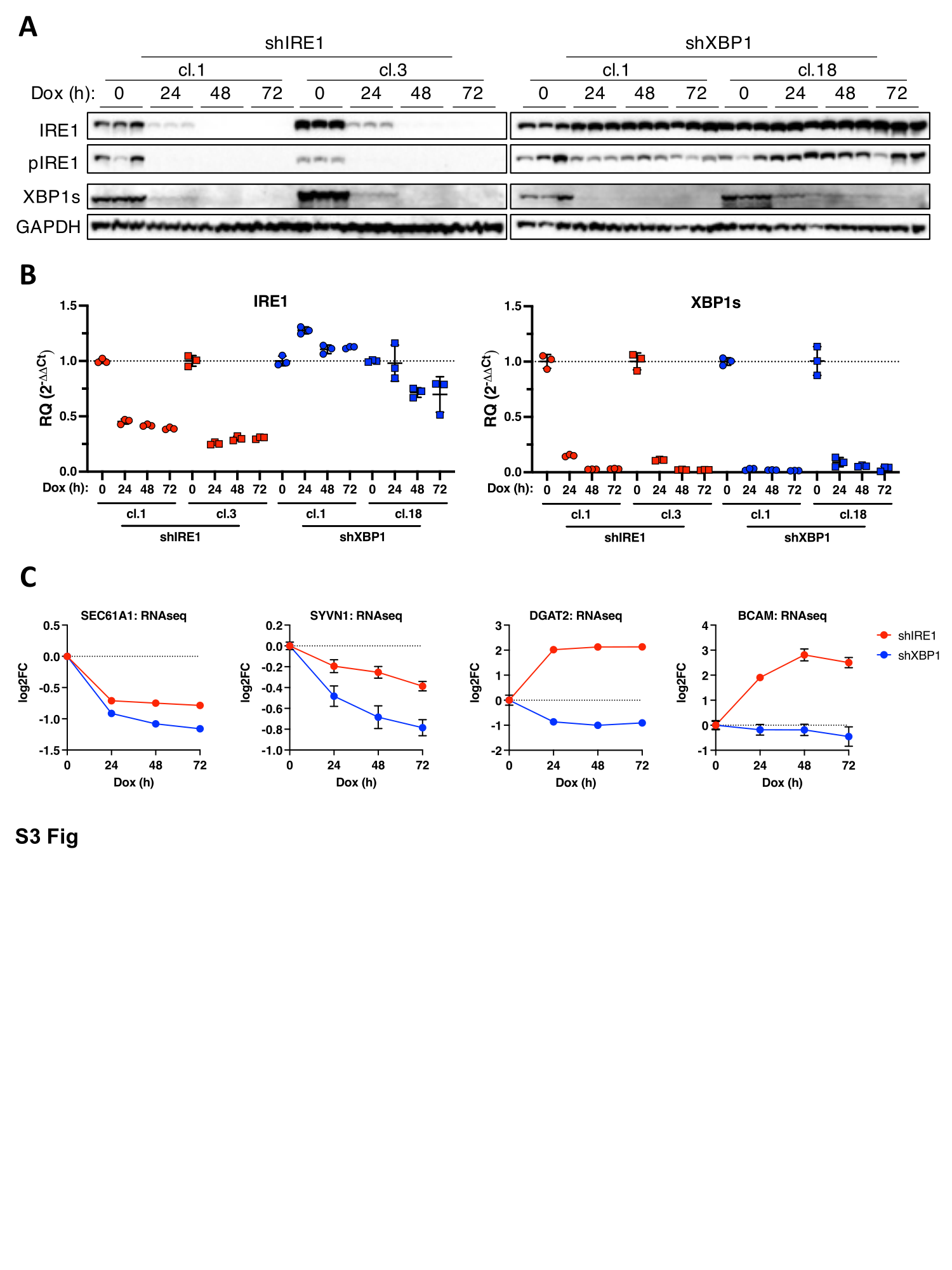

Supplement: S3 Fig — (A) RNA sequencing sample validation. AMO1 shIRE1 cl.1 or cl.3 cells and shXBP1 cl.1 and cl.18 cells were incubated for the indicated time with Dox (0.2 μg/ml) in triplicates and analyzed by IB. (B) Cells as in A were analyzed by RT-qPCR. Data points represent one biological replicate. Error bars represent SD. (C) Effect of IRE1 or XBP1 knockdown on mRNA expression of select XBP1s- and RIDD-target genes. Cells as in A were analyzed by bulk RNA sequencing (RNAseq) for mRNA expression of the XBP1s targets Sec61A1 and SYVN1 or the RIDD targets DGAT2 and BCAM. (D) Row-clustered heatmap of the scaled mRNA expression by bulk RNAseq of genes involved in the S phase of the cell cycle from RNA sequencing of cells as in A, used to calculate the S phase score in Fig 3B. (E) Row-clustered heatmap of the scaled mRNA expression by bulk RNAseq of genes involved in the G2 and M phases of the cell cycle from RNA sequencing of cells as in A, used to calculate the G2/M phase score in Fig 3C. (F) PROGENy pathway analysis of the effect of IRE1 or XBP1 knockdown on mRNA expression. PROGENy scaled pathway score heatmap depicting p53 as one of the most differentially altered pathways upon IRE1 versus XBP1s knockdown for samples depicted in Fig 3A. (G) Effect of IRE1 or XBP1 knockdown on mRNA expression of p53 target genes. Row-clustered heatmap depicting scaled mRNA expression by RNA sequencing of the top 100 TP53 pathway response genes for samples shown in Fig 3A, given the PROGENy model. (H) Effect of IRE1 or XBP1 knockdown on p53 cleavage. AMO1 shIRE1 cl.1 or shXBP1 cl.1 cells were incubated for the indicated time with Dox (0.2 μg/ml) and QVD (30 μM) and analyzed by IB. Representative blot of 3 independent experiments shown. (I) Effect of IRE1 or XBP1 knockdown on CDKN1B/p27 protein levels. Data depicted are from the proteomics analysis described in Fig 3F. Data points are mean ± SE for all biological and technical replicates normalized to t = 0 h for each cell line. (J) Effect of IRE1 [file pbio.3003920.s001.tiff]
