# Supplementary material for: Editorial Note: A nonenzymatic dependency on inositol-requiring enzyme 1 controls cancer cell cycle progression and tumor growth
Source: PLoS Biol. 2026 Jul 29;24(7):e3003920. doi: 10.1371/journal.pbio.3003920 (PMC13419169; doi:10.1371/journal.pbio.3003920)
Supplement: S1 File — This diagram was also published in [2]. (PDF) [file pbio.3003920.s002.pdf]

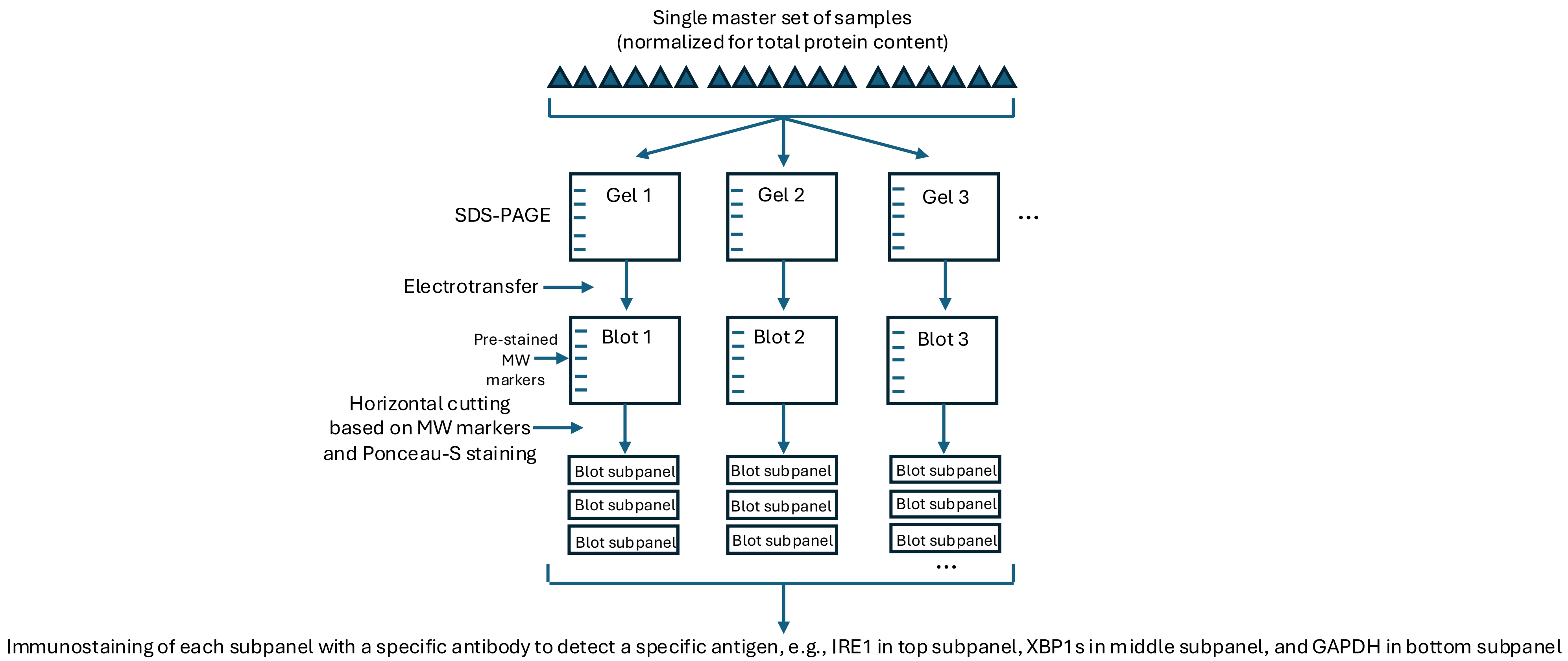
Single master set of samples
(normalized for total protein content)
SDS-PAGE
Gel 1
Gel 2
Gel 3
...
Electrotransfer
Blot 1
Blot 2
Blot 3
Pre-stained MW markers
Horizontal cutting based on MW markers and Ponceau-S staining
Blot subpanel
Blot subpanel
Blot subpanel
Blot subpanel
Blot subpanel
Blot subpanel
Blot subpanel
Blot subpanel
Blot subpanel
...
Immunostaining of each subpanel with a specific antibody to detect a specific antigen, e.g., IRE1 in top subpanel, XBP1s in middle subpanel, and GAPDH in bottom subpanel
